# Nasal-type extranodal natural killer/T-cell lymphoma presenting with a mass on the buttock: A case report

**DOI:** 10.1097/MD.0000000000018260

**Published:** 2019-12-10

**Authors:** Shuzhong Liu, Xi Zhou, An Song, Zhen Huo, Yipeng Wang, Yong Liu

**Affiliations:** a Department of Orthopedic Surgery, Peking Union Medical College Hospital, Peking Union Medical College and Chinese Academy of Medical Sciences; b Department of Endocrinology, Key Laboratory of Endocrinology, National Health and Family Planning Commission; c Department of Pathology, Peking Union Medical College Hospital, Chinese Academy of Medical Science & Peking Union Medical College, Beijing, China.

**Keywords:** autologous hematopoietic stem cell transplantation, chemotherapy, diagnosis, extranodal lymphoma, mass on the buttock, nasal-type natural killer/T-cell lymphoma, surgical treatment

## Abstract

**Rationale:**

Nasal-type extranodal natural killer (NK)/T-cell lymphoma is a highly unusual disease with no standard curative managements yet. Our focus is to emphasize a very rare case of nasal-type extranodal NK/T-cell lymphoma with onset of the mass on the buttock successfully operated by combination of surgical excision together with chemotherapy. The management of these unique cases is of great clinical guiding significance.

**Patient concerns:**

A 20-year-old woman presented with a 2-month history of continuous and progressive severe pain on her left buttock. Since December 2017, the patient developed nasal congestion, accompanied with mild pain in the left eye, and new subcutaneous nodules on both cheeks.

**Diagnosis:**

Postoperative pathology confirmed the diagnosis of extranodal NK/T cell lymphoma. This is an extremely rare presentation of nasal-type NK/T-cell lymphoma.

**Interventions:**

The patient underwent enlarged resection of the tumor on the buttock. After the diagnosis of extranodal NK/T-cell lymphoma was established, the patient received chemotherapy and autologous hematopoietic stem cell transplantation.

**Outcomes:**

The patient's symptoms improved significantly after the surgery, and the postoperative period was uneventful at the 1-year follow-up visit. There were no complications associated with the operation and adjuvant therapies during the follow-up period.

**Lessons:**

Taken together, the lesion's clinical features, imaging results, and pathological characteristics are unique. Extranodal NK/T-cell lymphoma, although rare, should be part of the differential diagnosis when the patient presents with the mass on the buttock. We recommend enlarged excision of the extranodal lymphoma. Combined of surgical excision of the extranodal lymphoma, chemotherapy and autologous hematopoietic stem cell transplantation are good choice for proper treatment.

## 1 Introduction

Nasal-type extranodal natural killer (NK)/T-cell lymphoma (ENKTL) is a rare lymphoma associated with Epstein–Barr virus (EBV), which is more common in East Asia than in the Western countries.^[1–3]^ ENKTL is an aggressive lymphoma preferentially occurring at unusual sites, including the central nerve system, skin, nasopharynx, and other areas, and it may deteriorate rapidly despite active treatment.^[2]^ Cutaneous lesions are among the rarest that are occasionally observed as sites of ENKTL involvement, and extranodal involvement often leads to an unfavorable prognosis. However, we still know little about the underlying biological nature involved in the development and management of this disorder. Herein, we report a rapid progressing case of ENKTL with initial presentation of the mass on the buttock, and present the clinical, radiological, and histological features of the patient. Our focus is to emphasize the importance of considering extranodal lymphoma as a diagnosis and guiding the proper management strategy for these patients.

## 2 Case report

A 20-year-old female patient with severe pain on her left buttock was originally given treatment by another hospital since August 2017. Analgesic therapy was hence proceeded for pain-relieving purpose, but to little effect. Magnetic resonance imaging (MRI) of the hip revealed the lesion appeared hyperintense on T1-weighted image, T2-weighted image, and diffusion-weighted image, whose boundary was clear and the size was about 2.8 × 2.6 cm. Tissue biopsy was performed and the pathology result revealed the features of obvious cell pleomorphism; thus, malignancy was considered. With a rapidly increasing mass reaching the size of about 7.0× 6.0 cm after 1 month, accompanied by a worsened pain on her left buttock, the patient came for consultation in our institution in September 2017. In the medical journal of her current illness, she stated that she did not experience any fever, weekness, weight loss, sweating, or other B symptoms, nor was she ever been injured or having underlied diseases. No pertinent family history was identified, including, hypertension, cancer, and congenital birth difficulties.

**Figure 1:**
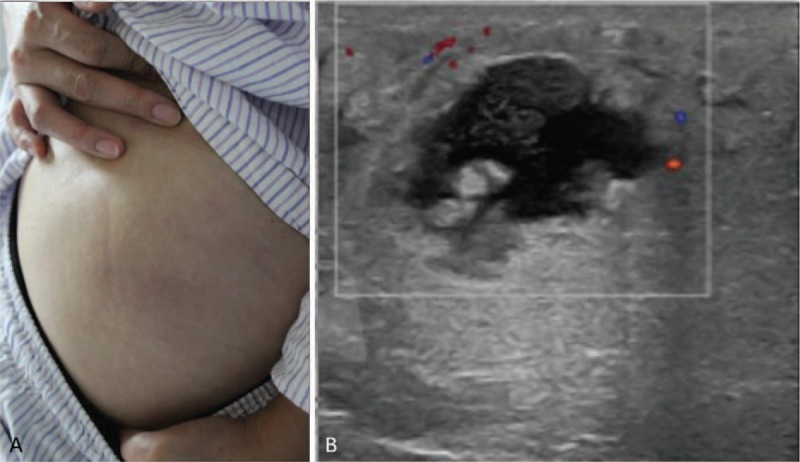
(A) Physical examination revealed a mass sized at 7.0 × 6.0 cm on her left buttock, accompanied by local red, swelling, and tenderness. (B) Ultrasonic examination of the mass disclosed the echo of the soft tissue layer is diffuse and the boundary changed to be unclear, and anechoic region was seen inside the mass.

**Figure 2:**
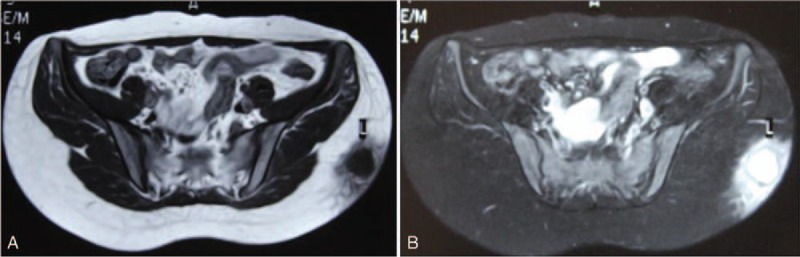
(A and B) Preoperative transverse magnetic resonance imaging scan revealing the soft tissue tumor on left buttock.

Physical examination revealed a mass sized 7.0 × 6.0 cm on her left buttock, accompanied by local red, swelling, and tenderness (Fig. 1A). Preoperative laboratory assessment was conducted, including routine laboratory tests (electrolytes, liver and kidney function tests, complete blood count), tumor markers, immunoglobulin M, immunoglobulin G, serum lactate dehydrogenase, and serum infectious indexs. Results confirmed that most indicators were within normal range. Ultrasonic examination disclosed the echo of the soft tissue layer on left buttock is diffuse and the boundary changed to be unclear, and anechoic region was seen inside the mass (Fig. 1B). MRI of the hip revealed the lesion with hyperintense signals on T1-weighted image and T2-weighted image, sizing about 6.8 cm × 5.9 cm, and the enhanced scan showed a circular strengthening and hyperintense signals on T1-weighted image, T2-weighted image of the surrounding tissue (Fig. 2A and B).

**Figure 3:**
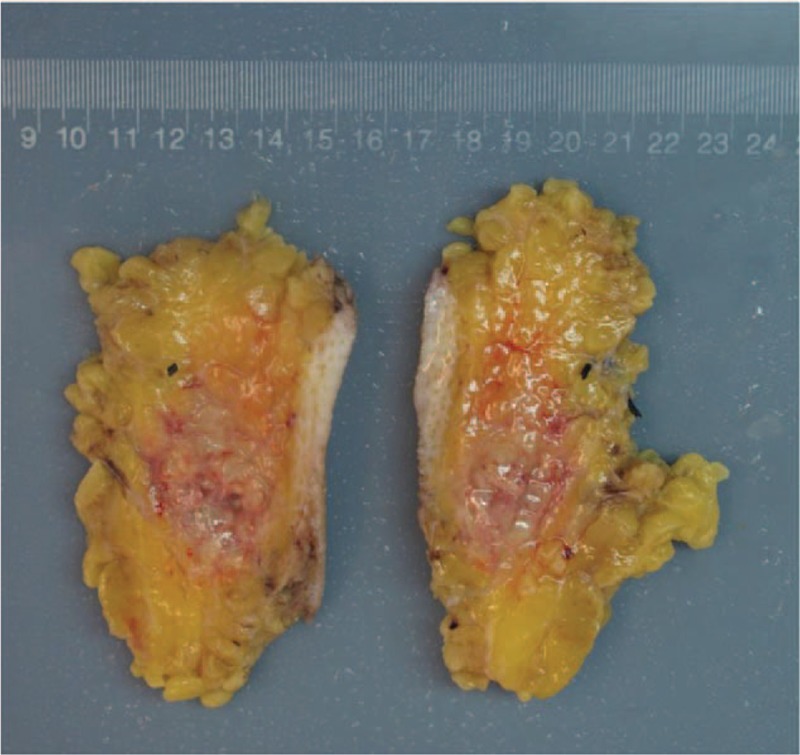
Intraoperative photography depicting the resected tumor.

**Figure 4:**
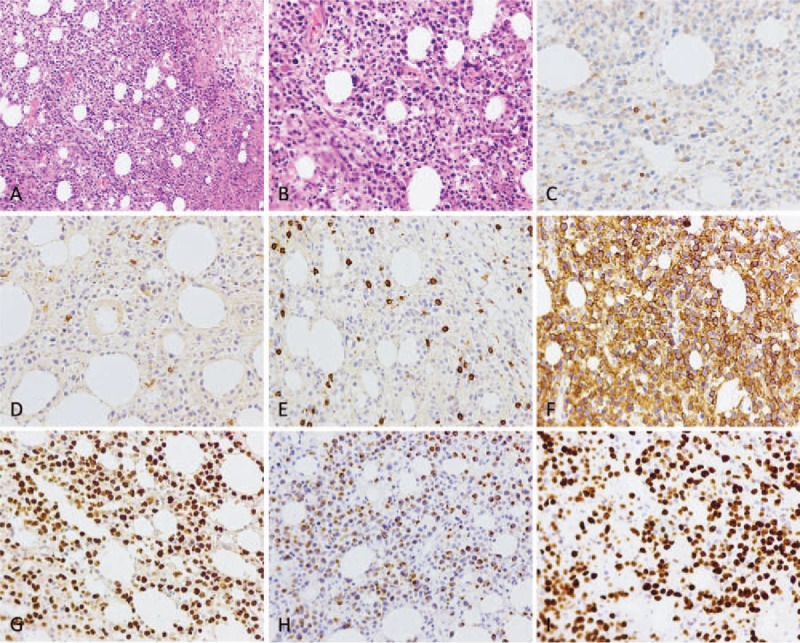
Pathologic histology of nasal-type extranodal natural killer/T-cell lymphoma. (A and B) Microphotography showing characteristic nests of tumor cells separated by vascular septa (Zellballen) with cells showing significant nuclear pleomorphism with prominent nucleoli (H&E, original magnification 100× and 200×). (C) CD3 immunostaining is strongly positive in the cells. (D–H) CD4, CD8, CD56, EBER ISH, TIA1 immunostaining shows strong, diffuse cytoplasmic staining in the tumor cells. (I) Ki-67 immunostaining shows 60% Ki-67-positive cells. Ki-67 staining is localized in the tumor nuclei.

After detailed evaluation, enlarged resection of the tumor on her left buttock was performed under general anesthesia. During the operation, it can be seen that the gray-white and tough mass was about 7 × 6 × 5 cm in size near the subcutaneous part of the superficial fascia, with unclear boundary and no capsule. The surrounding tissues were separated 4 cm away from the edge of the lesion, and the mass and surrounding tissues were completely excised and then sent for pathological examination (Fig. 3). The wound was immersed in 50-mg cisplatin and 300-mL hot distilled water for 10 minutes, and then washed with sufficient physiological saline for another 10 minutes. Histology revealed an extranodal NK/T-cell lymphoma and immunohistochemistry revealed neoplastic cells being CD3, CD4, CD56, TIA-1, S-100, CD20, CD38, CD68, CD8, SMA, Desmin, CD30, MPO positive with 60% Ki-67 positive nuclei. Moreover, the pathological result was positive for Epstein–Barr virus (EBV)-encoded small RNA in situ hybridization (EBER-ISH) (Fig. 4A–I).

**Figure 5:**
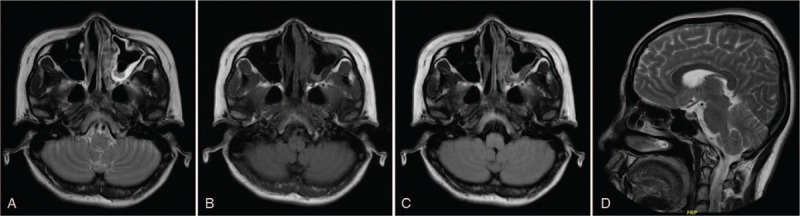
(A–D) Transverse and sagittal magnetic resonance imaging scan of the brain revealing abnormal signals in the left nasal cavity.

**Figure 6:**
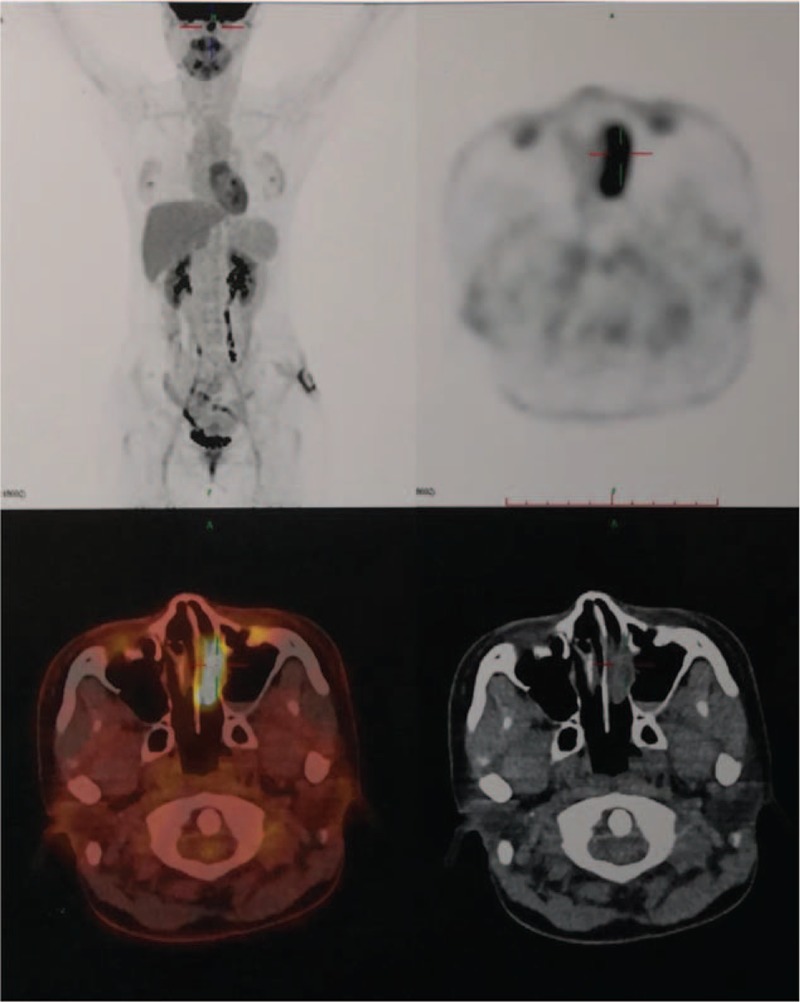
Positron emission tomography-computed tomography revealed abnormal high-intake in the left nasal cavity (1.2 × 3.3 × 1.4 cm, SUVmax 16.9).

**Figure 7:**
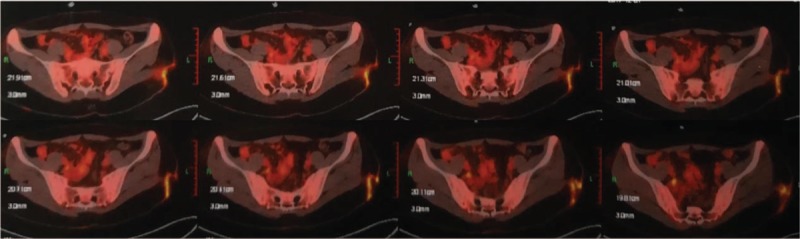
Positron emission tomography-computed tomography revealed irregular high-intake in the left hip.

Since December 2017, the patient developed nasal congestion, accompanied with mild pain in the left eye, and new subcutaneous nodules on her both cheeks. Cranial MRI showed abnormal density of soft tissue mass in her left nasal cavity (Fig. 5A-D). Positron emission tomography (PET)/computed tomography (CT) was performed and showed abnormal high-intake in the left nasal cavity (1.2 × 3.3 × 1.4 cm, SUVmax 16.9), multiple slightly higher metabolic nodules on the skin surface of frontal, maxilla, and left buttock (SUV 1.5–4.5) (Figs. 6 and 7). Consequently, a primary nasal-type extranodal NK/T—cell lymphoma, Ann Arbor stage IV A, International Prognostic Index score 2, was diagnosed via history taking, disease course, laboratory values, pathological studies, and fluorodeoxyglucose-positron emission tomography /computed tomography (FDG-PET/CT). An effective control with marked regression of the tumor was observed on radiography and FDG-PET/CT after o1 cycle of chemotherapy with CHOD (cyclophosphamide, epirubicin, vincrisin, and dexamethasone) and then four cycles of chemotherapy with GDP-ML (gemcitabine, cisplatin, methotrexate, dexamethasone, and pepsinase), followed by autologous hematopoietic stem cell transplantation. FDG-PET/CT demonstrated that the range of locally hypermetabolic lesions and the metabolic activity were both reduced significantly and the enlarged lymph nodes were not seen around the lesion, compared with multiple hypermetabolic lymph nodes revealed by FDG-PET/CT in December 2017. This is an extremely rare presentation of nasal-type NK/T-cell lymphoma. The 1-year follow-up visit found that the patient was doing well, with no evidence of local progress or distant metastases. There were no complications associated with the operation and adjuvant therapies during the follow-up period.

## 3 Discussion

Extranodal NK/T-cell lymphoma is a rare type of non-Hodgkin’ lymphoma (NHL) that accounts for 5%-18% of all NHLs.^[1–3]^ Nasal-type NK/T-cell lymphoma is a rare entity characterized predominantly by extranodal involvement and association with EBV infection.^[4,5]^ There are significantly regional and racial differences in its prevalence, and the cases in Asia, Mexico, and South America account for about 70% of all cases throughout the world.^[1–3,6–8]^ Although it may involve nasal cavity, upper respiratory tract, gastrointestinal tract, testes, brain, salivary glands, pancreas, soft tissues, adrenal glands, bone marrow, and other extranodal sites, patients with onset of cutaneous involvement is exceedingly rare in literature.^[1–5]^

ENKTL, nasal type, is deviated from either activated NK cells or cytotoxic T cells.^[1,2,9]^ It is an extremely rare and aggressive malignancy, and only a few cases have been documented so far.^[1,2,5,9–12]^ Patients may present with fever, cough, dyspnea, and other symptoms, which may be nonresponsive to antibiotics.^[9–12]^ Our reported case presented with severe pain and the mass on her left buttock; subsequently, the patient developed nasal congestion accompanied with mild pain in the left eye, and new subcutaneous nodules on both cheeks, which was consistent with extranodal NK/T-cell lymphoma.

The most common radiographical findings are the nodules or masses in tissues and organs.^[1–5,13–15]^ CT and MRI findings of the extranodal NK/T cell lymphoma usually vary and are nonspecific. In literature, these findings can be divided into 3 types—nodular or mass-like, mesenchymal-like, and pneumonia-like.^[13,16–19]^ Imaging studies including CT, MRI, bone scan, and PET/CT are nonspecific, making it difficult to differentiate ENKTL from other common disorders. However, imaging studies may play a crucial role in the accurate diagnosis and decision making of surgical intervention.^[20,21]^ In our patient, magnetic resonance imaging of the left buttock revealed the lesion appeared hyperintense on T1-weighted image, T2-weighted image, and diffusion-weighted image. Further PET/CT showed high-intake in the left nasal cavity (1.2 × 3.3 × 1.4 cm, SUVmax 16.9), and multiple slightly higher metabolic nodules on the skin surface of frontal, maxill and left buttock (SUV 1.5–4.5).

With the aid of morphological traits, immunohistochemistry, and EBER-ISH test, the final diagnosis of nasal-type ENKTL is established.^[22–24]^ The diagnosis of nasal-type ENKTL is on the basis of following features: lesions occur in the nose, skin, facial midline, lung, and other soft tissues or organs; ISH shows being positive for EBER; the lymphoma infiltrates in a diffuse pattern and is usually angiocentric and angiodestructive with coagulative necrosis and apoptotic bodies; cells are positive for CD2, CD3, CD56, TIA1, and other typical markers.^[1–5,22–26]^ This patient was positive for EBER, CD3, CD4, CD56, TIA-1, S-100, CD20, CD38, CD68, CD8, SMA, Desmin, CD30, MPO, with 60% Ki-67 positive nuclei, and the pathology results met the diagnostic criteria of nasal-type ENKTLs. Most patients with extranodal NK/T-cell lymphoma present with a mass or nodule; thus, they are often misdiagnosed as other lesions. Therefore, tissue biopsy is much needed for accurate diagnosis and proper treatment.^[25,26]^

Some previous retrospective studies have shown that the majority of ENKTLs has a poor prognosis; however, management of these unique cases has remained under evaluation, with no standard criteria to date.^[1–5,27–29]^ Correct diagnosis and timely treatment do have benefits for improving the prognosis. The optimal treatment has not been clearly established, although >70% of patients with ENKTLs received CHOP (cyclophosphamide, adriamycin, vincristine, and prednisone)-based chemotherapy and surgical resection in literature.^[28,29]^ During the last decade, first-line treatment for localized nasal-type ENKTL has changed from conventional anthracycline-containing chemotherapies to autologous hematopoietic stem cell transplantation (AHSCT) with chemoradiotherapy.^[29–31]^ High-dose chemotherapy with autologous hematopoietic stem cell transplantation for aggressive non-Hodgkin lymphoma is highly recommended in different settings: for patients with a chemosensitive relapse; for high-intermediate and high-risk patients as consolidation of a complete remission; for partial responders after first-line therapy.^[29–31]^ Although AHSCT may induce prolonged remissions and improve the prognosis, the exact role of transplantation in partial or complete responders is still uncertain due to the rarity of these patients. Therefore, more randomized controlled trials are needed for further confirmation of the conclusion. The recurrence rate for ENKTL is very high, and some patients might develop local recurrence, or distant metastasis soon after initial treatment.^[30,31]^

In conclusion, we present in this report a case with an extremely unusual occurrence of nasal-type ENKTL with rapidly increasing mass on the buttock and that was controlled by surgery, chemotherapy, and autologous hematopoietic stem cell transplantation. Such case has not been well defined in literature and it highlights the significance of early diagnosis and proper treatment for nasal-type ENKTL. Although uncommon, nasal-type ENKTL should be part of the differential when the patient presents with mass of unknown origin. Clinical symptoms are generally the result of the tumor burden, and pathological results remain the “criterion standard” for diagnosing nasal-type ENKTL. We recommend surgical excision of the extranodal lymphoma. Combined of surgical excision of the extranodal lymphoma and chemoradiotherapy with autologous hematopietic stem cell transplantation is a good choice for standard treatment. With a multidisciplinary team approach, proper planning, and adequate perioperative medical management, nasal-type ENKTL can be managed much more effectively.

## Acknowledgments

The authors thank their colleagues at the Department of Orthopaedic Surgery, Peking Union Medical College Hospital, Chinese Academy of Medical Sciences and Peking Union Medical College.

## Author contributions

**Conceptualization:** Shuzhong Liu, Xi Zhou, An Song, Yipeng Wang, Yong Liu.

**Funding acquisition:** Shuzhong Liu, Yipeng Wang, Yong Liu.

**Investigation:** Shuzhong Liu, Xi Zhou, An Song, Yong Liu.

**Project administration:** Shuzhong Liu, Yong Liu.

**Resources:** Shuzhong Liu, Xi Zhou, Zhen Huo, Yong Liu.

**Supervision:** Yipeng Wang, Yong Liu.

**Writing – original draft:** Shuzhong Liu, An Song, Yong Liu.

**Writing – review & editing:** Shuzhong Liu, Yipeng Wang, Yong Liu.
